# Magneto-Optical Spectroscopy of Short Spin Waves by All-Dielectric Metasurface

**DOI:** 10.3390/nano12234180

**Published:** 2022-11-25

**Authors:** Daria O. Ignatyeva, Vladimir I. Belotelov

**Affiliations:** 1 Russian Quantum Center, 121353 Moscow, Russia; 2 Photonics and Quantum Technologies School, Faculty of Physics, Lomonosov Moscow State University, 119991 Moscow, Russia

**Keywords:** magneto-optics, nanophotonics, polar Kerr effect, spin waves

## Abstract

The optical method of spin dynamics measurements via the detection of various magneto-optical effects is widely used nowadays. Besides it being a convenient method to achieve time-resolved measurements, its spatial resolution in the lateral direction is limited by a diffraction limit for the probe light. We propose a novel approach utilizing a Mie-resonance-based all-dielectric metasurface that allows for the extraction of a signal of a single submicron-wavelength spin wave from the wide spin precession spectra. This approach is based on the possibility of designing a metasurface that possesses nonuniform magneto-optical sensitivity to the different nanoscale regions of the smooth magnetic film due to the excitation of the Mie modes. The metasurface is tuned to be unsensitive to the long-wavelength spin precession, which is achieved by the optical resonance-caused zeroing of the magneto-optical effect for uniform magnetization in the vicinity of the resonance. At the same time, such a Mie-supporting metasurface exhibits selective sensitivity to a narrow range of short wavelengths equal to its period.

## 1. Introduction

Ultrafast manipulation of spins at the nanoscale without Joule losses [1,2] is crucial for many practical applications, including information processing [3,4,5,6] quantum computing [7], memory [8], and quantum networks [9]. Optical methods of ultrafast spin dynamics launching have recently been developed in various types of magnetic films [10,11] and nanostructures [12,13,14].

The optical method of spin dynamics measurements via the detection of various magneto-optical effects is widely used in pump-probe setups [11]. The main advantage of this method is the ability to obtain sub-picosecond temporal resolution [15], which is achieved by the controlled delay between the pump and the probe pulses. Moreover, spin-wave dispersion could be reconstructed using time-resolved magneto-optical measurements [16]. One may also benefit from the tunability of the magneto-optical measurements since the variation of the light wavelength, polarization, and distribution inside a magnetic film allows for the detection of various non-uniform spin-wave modes [12,17,18].

At the same time, the spatial resolution in the lateral direction provided by the magneto-optical method is limited by the diameter of the probe pulse due to a diffraction limit. This means that short spin waves with wavelengths smaller than the probe diameter are invisible to magneto-optical detection. Some efforts are made to overcome this limit, for example, via deposition of the nanoplasmonic structures on the top of the magnetic films [19]. However, this method provides sensitivity to all long-wavelength and a set of short-wavelength spin waves, and it is impossible to distinguish the measured wavelengths.

We propose a novel approach utilizing a Mie-resonance-based all-dielectric metasurface that allows for the extraction of a signal of a single submicron-wavelength spin wave from the wide spin precession spectra. This approach is based on the possibility of designing a metasurface that possesses nonuniform magneto-optical sensitivity to the different nanoscale regions of the smooth magnetic film due to the excitation of the Mie modes in Si nanoantennas deposited on its top. The system is tuned to be unsensitive to the long-wavelength spin precession, which is achieved by the optical resonance-caused zeroing of the magneto-optical effect for uniform magnetization in the vicinity of the resonance. Such resonant phenomena were earlier demonstrated for several types of nanostructures with localized plasmonic resonances [20,21], but applications of such types of structures for spin wave detection have not been studied yet. We show that, in addition to the lack of magneto-optical sensitivity to long-wavelength spin waves, such a structure has selective sensitivity to a narrow range of wavelengths equal to the structure period. Along with sensing and magnetometry [22,23,24,25,26,27], this is a promising direction of magnetic metasurface applications.

## 2. Magneto-Optical Spin-Wave Detection

Quantitative analysis and comparison of sensitivities of different magneto-optical detection schemes can be performed using the magnitude Φ of the magneto-optical response provided by a certain configuration under excitation of a spin wave with $\Lambda_{\mathrm{sw}}$ wavelength. Detection of the spin waves could be performed using various magneto-optical effects, so that Φ could represent the magnitude of different magneto-optical effects, such as Faraday rotation [12,13,16,28], the polar Kerr effect [29], magnetic linear birefringence, or the Cotton–Mouton effect [28,30]. In the present study, we focus on the configuration where Φ is the polar Kerr effect value measured in a typical pump-probe setup under the excitation of backward volume or surface spin waves in the in-plane magnetized film.

Obviously, the sensitivity of the setup to the excited spin wave depends on both spin-wave and light properties. The magneto-optical effect provided in a certain scheme used for detection can be roughly estimated using the following considerations. Each region of the uniformly magnetized film contributes ϕ(x,y,z) to the measured magneto-optical effect so that ∫ϕ(x,y,z)dxdydz=Φ(M=const). Thus, the magneto-optical effect produced in the presence of the spin wave with $M_{\mathrm{sw}}\left(x,y,z\right)$ spatial distribution profile will be defined as:

(1) $$\Phi \propto \int M_{\mathrm{sw}}\left(x,y,z\right)\phi \left(x,y,z\right)dxdydz.$$

Notice that all of the near-field effects arising from the interference or nanostructure deposition on the films or tailoring of the probe spatial profile are taken into account in the ϕ(x,y,z) term.

The contributions of the different film regions ϕ(x,y,z) to the magneto-optical effect measured in the structure are proportional to the spatial distribution of the light intensity I(x,y,z). Because polarization rotation is typically measured as the sum signal obtained from the entire area illuminated with a probe light, the beam spatial profile I(x,y) is involved in magneto-optical measurements. On the other hand, the I(z) distribution is important, for example, in the case of thick films [17] or in photonic crystals with inhomogeneous light distributions [31]. Taking into account these considerations, and generalizing the theory presented in [31] on the other types of nanostructure modes, one may come to a conclusion about the correlation of ϕ(x,y,z) and I(x,y,z).

**Figure 1:**
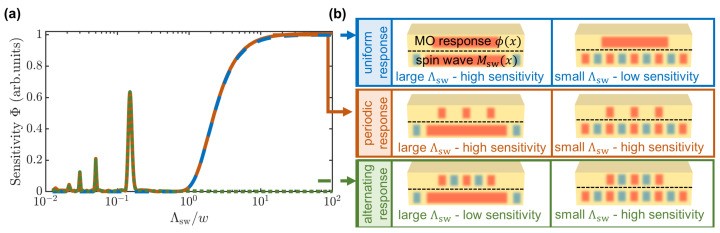
Comparison of the magneto-optical sensitivity to the spin waves of different wavelengths. (**a**) Estimation of the magneto-optical sensitivity calculated using Equation (1) in various cases: uniform ϕ(x) response of a bare film (blue line), localized sign-constant periodic ϕ(x) response (orange line) and alternating-sign ϕ(x) response (green line). (**b**) Scheme illustrating how the wavelength of the spin wave and type of ϕ(x) response cause different magneto-optical sensitivities. A more detailed explanation is provided in the text.

For a smooth magnetic film with a homogeneous magnetization $M\left(x,y\right)=M_{0}$, a 1D Gaussian probe pulse $I\left(x\right)\propto \exp\left(-4\ln\left(2\right)x^{2}/w^{2}\right)$ with a diameter *w* provides the sensitivity to various spin wavelengths equal to $\Phi \left(\Lambda_{\mathrm{sw}}\right)\propto 0.5\sqrt{\pi /\ln\left(2\right)}w$$\exp(-\pi^{2}w^{2}/\left(4\ln\left(2\right)\Lambda_{\mathrm{sw}}^{2}\right))$ (see Figure 1a, blue line). It means that such a probe is uniformly sensitive to the spin waves with large wavelengths $\Lambda_{sw}≳2w$ but cannot measure shorter wavelengths. The tighter the light is focused, the shorter the spin wavelengths that could be measured. However, the spot *w* cannot be smaller than the diffraction limit ∼1μm, so the practical limit of the spin-wave wavelengths that could be measured by such a scheme is also ∼1μm. The schemes illustrating how $M_{\mathrm{sw}}$ and ϕ distributions cause such sensitivity behaviour are presented in Figure 1b (top panel).

Efforts are being made to overcome this limit, and one of the ways to do this is the deposition of a nanostructure on the top of the magnetic film [19]. The magneto-optical sensitivity in this case may be roughly estimated in the following way. If a plasmonic structure made of periodically arranged nanoantennas is deposited on the top of the magnetic film, probe light concentrates predominantly under the nanoantennas, which significantly enhances the contributions ϕ(x) of these regions and simultaneously lowers the contributions of the other uncoated regions. In terms of the function describing magneto-optical contributions, this case corresponds to the constant-sign periodic ϕ(x) response. Therefore, such a structure becomes sensitive to the short spin waves with wavelengths $\Lambda_{\mathrm{sw}}=P/m$, where m∈N and *P* is the structure period. However, it still presumes sensitivity to the spin-wave wavelengths larger than the beam diameter $\Lambda_{\mathrm{sw}}≳2w$, as shown in Figure 1a (orange line) for ϕ(x)=0.5(sqw(x/P)+1), where sqw(x)=sgn(sin(2πx/P)) is a square wave function with *P* periodicity. Such a case is schematically depicted in Figure 1b (middle panel).

This long-wavelength sensitivity could be suppressed if a nanostructure with an alternating ϕ(x) response is created. The green line in Figure 1a and the bottom panel in Figure 1b show how the structure with alternating ϕ(x)=sqw(x/P) makes it possible to selectively detect spin-wave wavelengths equal to the structure period $\Lambda_{\mathrm{sw}}=P$. The following section is devoted to the design and physical principles of such structure’s operation on a basis of the Mie-resonant nanoantennas.

## 3. Magnetophotonic Spin-Wave Detection in Mie-Based Metasurface

An exciting possibility to get rid of the detection of background long-wavelength signals and to perform selective detection of the desired wavelength is provided by an all-dielectric metasurface. Mie-resonant nanoantennas of different shapes, among which are nanodisks, nanospheres, nanorods, nanostripes, etc., are commonly used to provide the resonant magneto-optical response of a metasurface [22]. For the sake of simplicity in analysis and potential fabrication, we consider a metasurface formed by a 1D grating of nanostripes of high-refractive-index material (for example, Si, GaP, and Ge) on the top of the magnetic film; however, similar results can be obtained for the nanoantennas of the other shapes. According to the Mie theory, such stripes produce resonances in the light scattering and absorption [32,33]. This scattered light experiences magneto-optical polarization rotation during its propagation through the magnetic film. The total magneto-optical response of a polar Kerr effect can therefore be represented as the sum of the ’Mie-scattered’ and reflected by the bare part of the film components. As the relative phases of these two components can be efficiently tuned by variation of the geometric parameters of the structure, i.e., the nanoantenna size, they may provide the opposite sign of the magneto-optical polarization rotation. In this case, the structure would be insensitive to the uniform magnetization and long-wavelength spin modes. In contrast to that, the structure would provide the magneto-optical response to the spin waves with a wavelength equal to the metasurface period, as suggested above.

**Figure 2:**
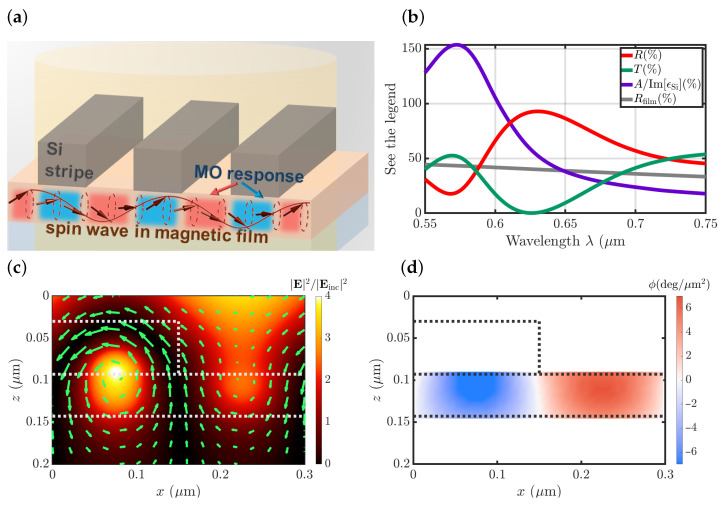
Optical properties of all-dielectric metasurface under the illumination of s-polarized light. (**a**) Scheme of the Mie-based structure and a magneto-optical short spin-wave spectroscopy. (**b**) Spectra of reflectance *R* (red line), transmittance *T* (blue line) and normalized absorption A/Im[εSi] (violet line) of the metasurface. Reflectance spectra of the same bare film are shown by the grey line $R_{\mathrm{film}}$. (**c**) Electromagnetic field distribution at λ=620 nm wavelength in the metasurface. ${\left|E\right|}^{2}$ is shown by colour, H-vector is shown by green arrows. Si stripe and magnetic film layer are shown by the grey dotted line. (**d**) Distribution of ϕ(x,z) at λ=620 nm wavelength in the metasurface shown as a false-colour plot.

In order to realize these conditions, the following structure was designed, see Figure 2a. Si stripes of $d_{\mathrm{Si}}=150$ nm width and $h_{\mathrm{Si}}=63$ nm thickness are deposited periodically with the period P=300 nm on the top of the iron-garnet magnetic film with $h_{m}=50$ nm thickness on a glass substrate. The details of numerical simulations presented in the manuscript are given in Appendix A.

The reflectance spectra of such metasurface exhibit a distinct resonant peak (Figure 2b) located at the slope of the Mie resonance, which, according to the Joule–Lenz law can be identified by the maxima of A/ImεSi∼|E|2 (Figure 2b). Electromagnetic field distribution has the electrodipole type in a Si stripe at the corresponding wavelength λ=620 nm (Figure 2c).

Due to a highly nonuniform distribution of the electromagnetic field and its polarization inside the magnetic film (Figure 2c), its different regions provide different contributions ϕ(x,z) to the total polar Kerr effect observed in a structure. These contributions were numerically analyzed (Figure 2d) by the calculation of the polar Kerr rotation of a metasurface with only a certain small region at (x,z) coordinates being magnetized, while the magnetization of the other film regions was set to zero. The integral of these contributions was checked to be equal to the polar Kerr effect observed for a fully magnetized film, ∫ϕ(x,z)dxdz=Φ(M=const).

**Figure 3:**
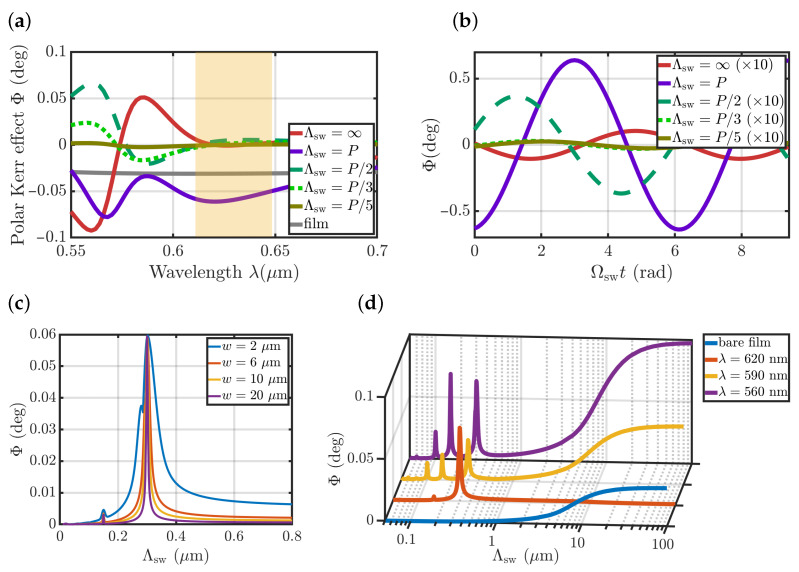
Magnetophotonic detection of the short spin waves. (**a**) Polar Kerr effect Φ spectra for different spin-wave wavelengths $\Lambda_{\mathrm{sw}}$ (see legend). (**b**) Polar Kerr effect Φ dependence on the phase of the spin wave at λ=620nm. (**c**) Polar Kerr effect Φ dependence on the laser beam width *w* for λ=620nm. (**d**) Sensitivity of the polar Kerr effect $\Phi (\Lambda_{\mathrm{sw}})$ measured at different wavelengths in the proposed metasurface and in the bare film (see the legend) by w=6μm beam width.

The spatial distribution of ϕ(x,z) (Figure 2d) shows that the two neighbouring regions, the one right below a Si stripe, and the other under its bare part, provide similar values but with opposite signs of ϕ(x,z). According to the theory presented above, this makes an integral in Equation (1) approximately zero for the large spin-wave wavelengths and uniform magnetization. The polar Kerr effect spectra of the uniformly magnetized metasurface indeed show nearly-zero polarization rotation at this light wavelength λ=620nm (Figure 3a, red line). In full accordance with the theoretical predictions, the polar Kerr effect in a structure with such ϕ(x,y) is high for a short-wavelength spin wave with $\Lambda_{sw}=P$ (Figure 3a, purple line) and nearly zero for the other $\Lambda_{sw}=P/m$ values. This is observed over a fairly broad frequency range of Δλ∼30 nm (Figure 3a, region marked as an orange rectangle), which is important for practical applications in pump-probe setups.

The total polar Kerr effect significantly depends on both the light and the spin-wave wavelength, as shown in Figure 3a. Furthermore, the value and sign of the polar Kerr effect depend on the phase of the spin wave $\Omega_{\mathrm{sw}}t$ as shown in Figure 3b as the spin wave $M_{z}$ component changes in each point of the structure $M_{\mathrm{sw}\;z}=M_{0}\sin\left(2\pi /\Lambda_{\mathrm{sw}}x+\Omega_{\mathrm{sw}}t\right)$. The values of these phases were chosen to provide maximal polar Kerr effect for each $\Lambda_{sw}$ shown in Figure 3a.

The polar Kerr effect observed in a metasurface for a certain spin-wave wavelength significantly depends on the width of the laser beam (Figure 3c). The wider the beam, the greater the impact of periodicity and ϕ(x) sign-changing behaviour, and the Mie-supporting metasurface detects a narrower range of spin waves.

Figure 3d shows the resulting sensitivity of the bare film and metasurface illuminated by different wavelengths of optical pulses to various spin-wave wavelengths. It is clearly seen that at the operating wavelength λ∼620nm the metasurface is selectively sensitive to the spin wave with a certain $\Lambda_{\mathrm{sw}}=P$ wavelength, while the other wavelengths, including the long ones and the uniform precession, do not make any contribution to the total polar Kerr effect. On the other hand, both bare film and periodic structure at the other wavelengths are sensitive to a wide range of long-wavelength spin waves.

**Figure 4:**
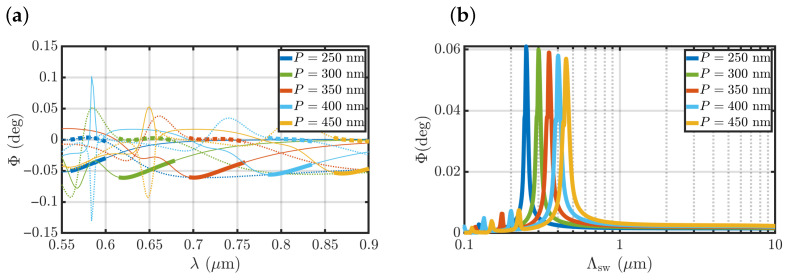
Tuning metasurface parameters to perform sensing. (**a**) Spectra of the polar Kerr effect Φ for the uniform magnetization $\Lambda_{\mathrm{sw}}=\infty$ (dotted line) and $\Lambda_{\mathrm{sw}}=P$ (solid line) calculated for several metasurface periods (see the legend). Regions corresponding to the selective MO sensing of $\Lambda_{\mathrm{sw}}=P$ are shown by thick lines. The Si nanostripe width was kept equal to the structure half-period $d_{\mathrm{Si}}=P/2$ while its thickness gradually increased from $h_{\mathrm{Si}}=52$ nm to $h_{\mathrm{Si}}=94$ nm with the period increase. (**b**) Sensitivity of the polar Kerr effect $\Phi (\Lambda_{\mathrm{sw}})$ for the metasurfaces with different periods (see the legend) for w=6μm beam width.

The magneto-optical response of a metasurface is determined by its parameters, including the size of nanostripes and their arrangement. Thus, it is possible to scale the metasurface to observe the above-described effect of selective magneto-optical sensing for a wide range of metasurface periods and the corresponding spin-wave wavelengths. Figure 4 shows how metasurfaces with different periods exhibit similar selective magneto-optical sensitivity to the certain spin-wave wavelength equal to its period.

The absolute value of the polar Kerr effect in the metasurface $\Phi \left(\Lambda_{sw}=P\right)=0.06^{\circ }$ is twice as high as that of a bare uniformly magnetized film $\Phi_{0}=0.03^{\circ }$ (Figure 3a, grey line), and the reflectance at this wavelength is also twice that of a bare film (Figure 2b). This means that the signal-to-noise ratio will be enhanced in the proposed metasurface compared to the one of the smooth film.

## 4. Conclusions

We propose a novel approach utilizing a Mie-resonance-based all-dielectric metasurface that allows for the extraction of a signal of a single submicron-wavelength spin wave from the wide spin precession spectra. This approach is based on the possibility of designing a metasurface that possesses nonuniform magneto-optical sensitivity to the different nanoscale regions of the smooth magnetic film due to the excitation of the Mie modes in the nanoantennas deposited on its top. The system is tuned to be unsensitive to the long-wavelength spin precession, which is achieved by the optical resonance-caused zeroing of the magneto-optical effect for uniform magnetization in the vicinity of the resonance. At the same time, such a Mie-supporting metasurface exhibits selective sensitivity to the short wavelengths equal to its period. As no special optical properties of the magnetic film are required except for its thickness and transparency, this method can be used for the spin-wave spectroscopy of a wide range of dielectric magnetic materials, including ferro-, ferri-, and antiferromagnetic materials.

## Data Availability

The data presented in this study are available on request from the corresponding author.

## Appendix A. Methods of Magneto-Optical Numerical Simulations

Electromagnetic simulations of the magneto-optical metasurface response were carried out by numerical solution of Maxwell equations using the RCWA (rigorous coupled-wave analysis) method [34,35]. The RCWA method provides both the far-field optical characteristics and near-field distributions of electromagnetic field for the structures with a 1D periodicity of permittivity and magnetization. Structure magnetization is taken into account as the non-diagonal elements of the permittivity tensor $\varepsilon_{xy}=ig_{z}∼M_{z}$.

For simulations, we used numerical values of dielectric permittivity and gyration of iron-garnet corresponding to the ones obtained in our previous experimental research [36,37]: $\varepsilon_{ii}=7.0134+0.0970i$, $\varepsilon_{xy}=0.01i$ in the vicinity of the light wavelength λ=600 nm. The dispersion from Ref. [38] providing the values $\varepsilon_{\mathrm{Si}}=15.5232+0.1571i$ and $\varepsilon_{{\mathrm{SiO}}_{2}}=2.13$ were used for the Si nanostripes and the glass substrate, correspondingly.

The magneto-optical contribution ϕ(x,z) function was calculated in a following way. The polar Kerr effect $\Phi (x_{0},z_{0})$ was calculated in a structure where the magnetization of a whole magnetic film is set to zero except a small region of Δx×Δz area located at $\left(x_{0},z_{0}\right)$ coordinate. After that, $\phi (x_{0},z_{0})$ was calculated as the ratio $\phi \left(x_{0},z_{0}\right)=\Phi \left(x_{0},z_{0}\right)/(\Delta x\Delta z$).

To describe the polar Kerr effect $\Phi (\Lambda_{\mathrm{sw}},\lambda ,w)$ observed in the case of a finite beam width and arbitrary spin-wave wavelength, we used a magneto-optical contribution ϕ(x,z) function calculated at the corresponding light wavelength λ and the Equation (1). Thus, $\Phi \left(\Lambda_{\mathrm{sw}},\lambda ,w\right)=\int m_{\mathrm{sw}}\;z\left(x,z\right)\tilde{I}\left(x,z\right)\phi \left(x,z\right)dxdz,$ where $m_{\mathrm{sw}\;z}=\sin\left(2\pi /\Lambda_{\mathrm{sw}}x+\Omega_{\mathrm{sw}}t\right)$ is the normalized z-component magnetization distribution due to a spin wave excitation, and $\tilde{I}\left(x,z\right)=I_{0}\exp\left(-4\ln\left(2\right)x^{2}/w^{2}\right)$ is the normalized light intensity distribution with a beam width *w* and $I_{0}={\left(\int_{-\infty }^{+\infty }\tilde{I}\left(x,z\right)dxdz\right)}^{-1}$.
